# Dehydroepiandrosterone (DHEA) Sensitizes Irinotecan to Suppress Head and Neck Cancer Stem-Like Cells by Downregulation of WNT Signaling

**DOI:** 10.3389/fonc.2022.775541

**Published:** 2022-07-13

**Authors:** Li-Jie Li, Chien-Hsiu Li, Peter Mu-Hsin Chang, Tsung-Ching Lai, Chen-Yin Yong, Sheng-Wei Feng, Michael Hsiao, Wei-Min Chang, Chi-Ying F. Huang

**Affiliations:** ^1^ Ph.D. Program in School of Dentistry, College of Oral Medicine, Taipei Medical University, Taipei, Taiwan; ^2^ Institute of Biopharmaceutical Sciences, National Yang Ming Chiao Tung University, Taipei, Taiwan; ^3^ Genomics Research Center, Academia Sinica, Taipei, Taiwan; ^4^ Department of Oncology, Taipei Veterans General Hospital, Taipei, Taiwan; ^5^ Faculty of Medicine, National Yang Ming Chiao Tung University, Taipei, Taiwan; ^6^ Division of Pulmonary Medicine, Department of Internal Medicine, Wan Fang Hospital, Taipei Medical University, Taipei, Taiwan; ^7^ Division of Oral and Maxillofacial Surgery, Department of Dentistry Wan Fang Hospital, Taipei Medical University, Taipei, Taiwan; ^8^ School of Dentistry, College of Oral Medicine, Taipei Medical University, Taipei, Taiwan; ^9^ Division of Prosthodontics, Department of Dentistry, Taipei Medical University Hospital, Taipei, Taiwan; ^10^ Department of Biochemistry, College of Medicine, Kaohsiung Medical University, Kaohsiung, Taiwan; ^11^ School of Oral Hygiene, College of Oral Medicine, Taipei Medical University, Taipei, Taiwan

**Keywords:** dehydroepiandrosterone, head and neck squamous cell carcinoma, WNT, stemness, irinotecan

## Abstract

**Purpose:**

Current treatment options for head and neck squamous cell carcinoma (HNSCC) are limited, especially for cases with cancer stem cell-induced chemoresistance and recurrence. The WNT signaling pathway contributes to maintenance of stemness *via* translocation of β-catenin into the nucleus, and represents a promising druggable target in HNSCC. Dehydroepiandrosterone (DHEA), a steroid hormone, has potential as an anticancer drug. However, the potential anticancer mechanisms of DHEA including inhibition of stemness, and its therapeutic applications in HNSCC remain unclear.

**Methods:**

Firstly, SRB assay and sphere formation assay were used to examine cellular viability and cancer stem cell-like phenotype, respectively. The expressions of stemness related factors were measured by RT-qPCR and western blotting. The luciferase reporter assay was applied to evaluate transcriptional potential of stemness related pathways. The alternations of WNT signaling pathway were measured by nuclear translocation of β-catenin, RT-qPCR and western blotting. Furthermore, to investigate the effect of drugs *in vivo*, both HNSCC orthotopic and subcutaneous xenograft mouse models were applied.

**Results:**

We found that DHEA reduced HNSCC cell viability, suppressed sphere formation, and inhibited the expression of cancer-stemness markers, such as BMI-1 and Nestin. Moreover, DHEA repressed the transcriptional activity of stemness-related pathways. In the WNT pathway, DHEA reduced the nuclear translocation of the active form of β-catenin and reduced the protein expression of the downstream targets, CCND1 and CD44. Furthermore, when combined with the chemotherapeutic drug, irinotecan (IRN), DHEA enhanced the sensitivity of HNSCC cells to IRN as revealed by reduced cell viability, sphere formation, expression of stemness markers, and activation of the WNT pathway. Additionally, this combination reduced *in vivo* tumor growth in both orthotopic and subcutaneous xenograft mouse models.

**Conclusion:**

These findings indicate that DHEA has anti-stemness potential in HNSCC and serves as a promising anticancer agent. The combination of DHEA and IRN may provide a potential therapeutic strategy for patients with advanced HNSCC.

## Introduction

Head and neck squamous cell carcinomas (HNSCCs) are a group of malignancies that arise from transformed cells of the oral cavity, oropharynx, larynx, or hypopharynx mucosa. HNSCC is the sixth most common cancer worldwide. Approximately 650,000 new cases of HNSCC are diagnosed every year, and it accounts for about 5% of all cancer-related deaths (1, 2). The standard treatment for HNSCC includes surgery, radiotherapy, chemotherapy, and combinations of these modalities. However, the survival rate of patients with HNSCC remains low because of drug resistance, tumor metastasis, and recurrence (3). Therefore, it is critical to understand the mechanisms of local recurrence, metastasis, and resistance that may significantly improve the treatment outcomes of patients with HNSCC.

Cancer stem cells (CSCs) are a subpopulation of cancer cells that possess self-renewal capacity and pluripotency. CSCs are involved in tumor development, cell proliferation, and metastasis, and are the key “seeds” for tumor initiation, metastasis, and resistance to chemo- and radiotherapies (1, 3–5). These processes are regulated by several key transcription factors involved in cancer stemness and sphere formation, such as OCT4, Nanog, SOX2, KLF4, and MYC. Additionally, many signaling pathways, such as the WNT and Notch pathways, also contribute to the development of cancer stemness (6–10).

The WNT signaling pathway involves in cell proliferation, survival, and progression, and influences the self-renewal of stem cells under physiological and pathological conditions (11, 12). Upon activation of the WNT pathway, unphosphorylated β-catenin translocates into the nucleus and subsequently triggers TCF/LEF-mediated transcription of downstream genes, such as *CCND1*, *MYC*, and *CD44*. Dysregulation of the WNT/β-catenin signaling pathway is strongly associated with tumorigenesis and progression by maintaining cancer stemness (13). Recent studies have focused on the therapeutic potential of agents targeting WNT signaling for cancer treatment in mono- or combination therapy (14).

Irinotecan (IRN) is a topoisomerase I inhibitor that has anticancer activity in solid tumors, such as metastatic colorectal and lung cancer (15–17). IRN showed some clinical benefit in recurrent or metastatic HNSCC (R/M HNSCC) (18–20). IRN is a prodrug that is converted into the active metabolite SN-38 by carboxylesterase (CES) 1 or 2 (17). *CES1* was found to be a poor prognostic marker for HNSCC in TCGA HNSCC cohort (21). It was upregulated in patients with poor prognosis and represented a good therapeutic target for IRN therapy (16, 17, 21). IRN mono- and combination therapies with other chemotherapeutic agents have been shown to improve the treatment response in cancer patients (19, 20, 22). Murphy et al. conducted a Phase II study of irinotecan in patients with R/M HNSCC, the cohort 1 including 22 patients received irinotecan 125 mg/m^2^/week for 4 weeks followed by a 2-week rest. Due to the excessive toxicity among cohort 1, the 16 patients from cohort 2 were given the reduced dosage of irinotecan at 75 mg/m^2^/week for 2 weeks followed by 1-week rest. Toxicity and response among two cohorts to treatments were assessed using standard criteria (20, 23). Overall response rate is defined as the proportion of patients who have a partial or complete response to therapy (23, 24). According to National Cancer Institute (NCI) (website: https://www.cancer.gov/publications/dictionaries/cancer-terms), survival rate is referred to the percentage of people in a study or treatment group who are still alive for a certain period of time after they were diagnosed with or started treatment for a disease, such as cancer. Murphy et al. study displayed that IRN showed a modest overall response rate of 21.2% (95% confidence interval [CI] = 9%-38.9%) for 33 evaluable patients at both dose levels (20). Median survival for all evaluable patients was 214 days (95% CI = 146-365 days) with a 1-year survival rate of 30.2% (20). Response to IRN and its toxic side effects appeared to be dose-dependent (20). Furthermore, the combination of IRN with cisplatin showed synergistic anticancer effect in a phase II trial (19) and the cisplatin/tegafur-uracil (UFUR)/irinotecan triple combination therapy demonstrated a moderate response in patients with R/M HNSCC (18). Toxicity to patients was tolerable, and the quality of life of the patients improved (18). However, IRN also induces side effects, such as diarrhea and neutropenia, which can be resolved by optimizing the treatment dosage or increasing the target specificity.

Dehydroepiandrosterone (DHEA) is an endogenous steroid precursor hormone. In humans, DHEA is produced in the brain, adrenal cortex, gonads, and gastrointestinal tract (25), and is stored in its sulfated form, DHEA sulfate (DHEA-S) (26). DHEA and DHEA-S are both the most abundant steroids in the human serum and are precursors of sex hormones, such as estrogen and androgen. Recently, DHEA has been reported to have several beneficial effects such as anti-obesity, hypoglycemia, anti-atherosclerosis, anti-aging, and memory-enhancing effects (27–29). Moreover, DHEA has anticancer effects *in vitro* and *in vivo* in several cancer types, including breast (30–32), hepatoma (27), myeloma (33), leukemia (34), colon adenocarcinoma (35), pancreatic cancer (36) and cervical cancer (37). In breast cancer, DHEA inhibited cell proliferation and metastatic processes, such as migration, invasion, and epithelial mesenchymal transition (EMT), and decreased spheroid size (30–32). In addition, in human sphere mesenchymal stem cells, DHEA suppressed stem cell gene expression (38) which suggests that DHEA may have the ability to suppress CSCs. However, the effect of DHEA on HNSCC, especially cancer stem cell-like traits, remain unclear. Here, we investigated the anti-tumor and anti-stemness potential effects of DHEA, as well as the efficacy of its combined use with IRN against HNSCC.

## Methods

### Cell Lines and Cell Culture

The human HNSCC cell line, CAL 27, was obtained from the American Type Culture Collection (ATCC, USA), and HSC-3 and SAS were obtained from the Japanese Collection of Research Bioresources Cell Bank (JCRB, Japan). The human oral fibroblasts (HOF) were obtained from the ScienCell Research (USA). The lentivirus packaging cell line human embryonic kidney (HEK)-293T was also obtained from the ATCC. All cell lines were cultured in standard medium according to the manufacturer’s instructions containing 10% fetal bovine serum (FBS; Gibco, USA), 1% L-glutamine (Gibco, USA), and antibiotics (penicillin and streptomycin; Gibco, USA), and maintained in a humidified atmosphere of 5% CO_2_ at 37°C.

### Chemical Compounds

Trans-dehydroepiandrosterone (DHEA) (Sigma #D4000) was dissolved in dimethyl sulfoxide (DMSO) and maintained in 1% DMSO in the medium during *in vitro* drug treatment at 0-400 µM. Irinotecan (IRN) used for *in vitro* studies was purchased from Sigma (#I1406) and treated with cells from 0-10 µM. Campto^®^ (irinotecan hydrochloride trihydrate) used for animal administration was obtained from Pfizer. For *in vivo* experiment, DHEA and Campto^®^ were given at 10 mg/kg and 50 mg/kg, respectively, *via* intraperitoneal (IP) injection. The further details were described in the figure legends. The chemical compounds and reagents used in this study are listed in **Supplementary Table 1**. During the drug treatment, control (vehicle) groups were maintained in 1% DMSO in the medium, which was the same condition as DHEA treatment.

### Sulforhodamine B (SRB) Assay and Synergistic Effect Assessment

Cells were plated at 2000 cells/well in a 96-well microplate. Following drug treatments for the desired periods, cells were fixed with 10% trichloroacetic acid (w/v) for 1 h at 4°C, washed with water, and air-dried. SRB solution (0.4% [w/v] in 1% acetic acid) was used to stain the cells for 1 h and then 1% acetic acid was used to wash and remove the excess dye. After adding 20 mM Tris-base, the optical density (OD) of the protein-bound dye was measured at 540 nm to obtain the absorbance. Cell viability was normalized to the control, and the IC_50_ was calculated using GraphPad Prism 7 software. The synergistic effect assessment was performed by CompuSyn software (https://www.combosyn.com/) according to the user instruction. The resulting combination index (CI) theorem of Chou-Talalay offers a quantitative definition for additive effect (CI = 1), synergism (CI < 1), and antagonism (CI > 1) in drug combinations (39).

### Sphere Formation Assay

Sphere formation assay was performed as described previously (40). Briefly, cells were incubated with serum-free medium supplemented with 20 ng/ml of bFGF (PeproTech #100-18B), EGF (PeproTech #AF-100-15), and 1× B27 supplement (Gibco #17504044) in a humidified 5% CO_2_ atmosphere at 37°C. Then the cells were co-incubated with drugs in ultra-low attachment 6-well plates (Corning) at a density of 5000 cells/well. The images of spheres were captured using a phase contrast microscope (Leica), and the sphere size was determined using ImageJ software. To quantify the sphere size, we drew a line and set as the known distance according to the scale bar from pictures by using “Analyze” and “Set scale” from ImageJ. Then, we drew lines equal to each sphere and then conducted to “Measure” from ImageJ. Finally, the results of measured length were further used to statistical analysis.

### Isolation of Nuclear Extract

Nuclear and cytosolic extracts were isolated from cells using the rapid, efficient and practical (REAP) method (41). Briefly, following drug treatment, cells were scraped with cold phosphate buffered saline (PBS) and suspended in ice-cold 0.1% NP-40. After pipetting and centrifugation, half of the supernatant was transferred to a new tube and diluted with 4X SDS sample buffer, which was the cytoplasmic fraction. The remaining cell pellet was washed twice with ice-cold 0.1% NP-40 and resuspended with 1X SDS sample buffer diluted in 0.1% NP-40, which constituted the nuclear fraction. To detect protein expression in the fractions, the cytoplasmic fraction and the nuclear fraction from each treatment were conducted western blotting assay as described in next section. α-tubulin was used as a cytoplasmic control; lamin A/C was used as a nuclear fraction control.

### Western Blotting Assay

After drug treatment, cells were lysed, and protein concentration was measured using the Bradford assay (Thermo). Protein lysates (30 μg) were separated by 10% sodium dodecyl sulfate polyacrylamide gel electrophoresis (SDS-PAGE) and then electro-transferred to 0.45 μM polyvinylidene difluoride (PVDF) membranes (Millipore). After blocking with 5% milk in Tris-buffered saline containing Tween-20 (TBST) for 1 h, the membranes were incubated with primary antibodies at 4°C overnight followed by incubation with the corresponding secondary antibody for 1 h. The expression signals were visualized using the Immobilon Western Chemiluminescent HRP Substrate (Millipore #WBKLS0500) and detected using the Fujifilm LAS4000 luminescent image analysis system. Protein levels were quantified using ImageJ, and the expression was normalized to that of the internal control (β-actin). The antibodies used in this study are listed in **Supplementary Table 2**.

### Establishment of Stable Cells and Reporter Assay

The pGreenFire TCF/LEF (T cell factor/lymphoid enhancer factor), Nanog, OCT4, and Notch1 reporter lentivectors were purchased from System Biosciences. Pseudo reporter viruses were produced as described in our previous study (42). Briefly, HEK293T cells were co-transfected with reporter lentivectors and packaging plasmids MD2G and pCMV-dR8.91 (RNAiCore, Taiwan). After 48 h of transfection, viral supernatants were collected and added to the culture medium of target cells along with polybrene (Sigma). To obtain stable cell lines, the target cells were selected in puromycin (1 μg/mL, Invitrogen) for 48 h. To assess the effect of DHEA on TCF/LEF, Nanog, OCT4, and Notch1-regulated transcription, the stable cells were treated with the drugs, and then promoter activity was measured using ONE-Glo Luciferase Assay System (Promega).

### Reverse Transcription and Real-Time PCR (RT-qPCR) Assay

Total RNA was extracted from the cells using the TRIzol method (Invitrogen #15596026). The total RNA (2 μg) was used as a template for reverse transcription performed with a SuperScript III kit (Invitrogen). The cDNA was subjected to RT-qPCR in triplicate using Omics Green qPCR Master Mix and Gunster MB-P08A 8-strip PCR tubes (Gunster Biotech Inc., Taiwan). The primers used are listed in **Supplementary Table 3**. The relative expression was obtained using the comparative Ct method after normalization to the expression of *GAPDH* in the StepOne™ Real-Time PCR System.

### *In Vivo* Experiments

All animal experiments were performed in strict accordance with the guidelines for the Care and Use of Laboratory Animals of the National Institutes of Health (NIH). The animal experimental protocol was approved by the Institutional Animal Care and Use Committee of *Academia Sinica* (Taipei, Taiwan; protocol no.: ASIACUC-R19-07-1329). Male NOD.CB17-Prkdcscid/NcrCrl (Nod-SCID) mice aged 5–6 weeks were used for all the experiments. To evaluate the *in vivo* tumorigenicity and anti-stemness ability, 3 × 10^6^ CAL 27 cells or 1000 FACS sorted CD44^+^/CD133^+^ CAL 27 stem-like cells resuspended in PBS were subcutaneously inoculated into the right flank of the mice. For the HNSCC orthotopic model (43), CAL 27 luciferase-expressing cells (5 × 10^5^) resuspended in PBS were injected into the buccal submucosa of mice. *In vivo* tumors were imaged using the IVIS Imaging System (Caliper Life Sciences), and the signal intensity of luciferase expression was measured. Drugs were administered *via* intraperitoneal injection twice per week. Tumor growth and body weights were measured once a week. To determine the tumor formation frequency of mouse models bearing CAL 27 stem-like cells, the formed tumor was examined by autopsy after eight weeks of standard DHEA treatment regimen.

### Hematoxylin and Eosin (H&E) and Immunohistochemical (IHC) Staining and Analysis

Tumor sections were formalin-fixed and paraffin embedded. H&E or IHC staining was performed using a Discovery XT automated immunostainer (Ventana Medical System). After dewaxing, deparaffinization, and rehydration, Tris-EDTA buffer was used for antigen retrieval. The sections were immunostained for PCNA (GTX #100539, 1:500, GeneTex, USA) and Ki67 (Dako #M7240, 1:150, DAKO/Agilent, Santa Clara, CA), and subsequently counterstained with hematoxylin.

### Statistical Analysis

All statistical analyses were performed using the Student’s one-tailed *t-*test using Prism 7 software (GraphPad Software Inc., La Jolla, CA, USA). Data are presented as the mean ± standard deviation (SD) or standard error of mean (SEM) from independent experiments. Statistical significance was set at *p *< 0.05.

## Results

### DHEA Showed Anticancer Effect and Suppressed Stemness Potential of HNSCC Cells

**Figure 1:**
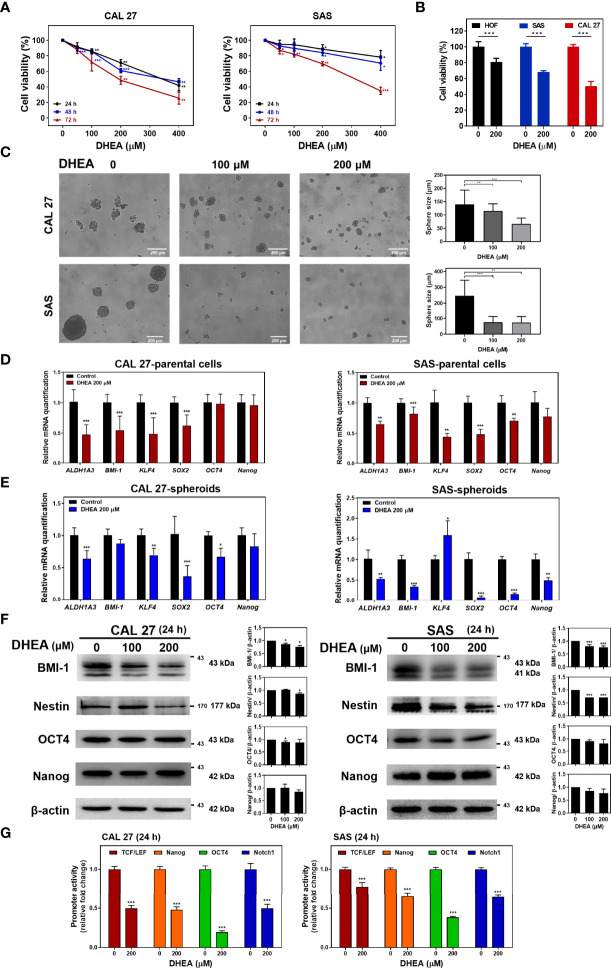
DHEA showed anticancer effect by reducing sphere size, expression of stemness markers, and transcriptional activity of related proteins in HNSCC cells. **(A)** HNSCC cells were treated with 0, 50, 100, 200, and 400 μM DHEA for 24, 48 and 72 h Cell viability was determined by SRB assay. **(B)** HNSCC cells and HOF were treated with 0 and 200 μM DHEA for 72 h. Cell viability was determined by SRB assay. **(C)** Sphere formation assay results showing CAL 27 and SAS cells incubated with 0, 100, and 200 μM DHEA for 20 days. Scale bar: 200 µm **(D, E)** RT-qPCR results showing mRNA level of stemness markers in CAL 27 and SAS parental cells **(D)**, and spheroids **(E)** after treatment with 200 μM DHEA for 6 h **(F)** Western blot analysis showing expression of stemness marker in CAL 27 and SAS cells after DHEA treatment for 72 h. *Left*, representative western blots of three independent experiments are shown. *Right*, bar charts represent the quantitation of three independent experiments. **(G)** Luciferase reporter assay showing transcriptional activity of stemness-related markers, including TCF/LEF (WNT), Nanog, OCT4, and Notch1 in CAL 27 and SAS cells after treatment with 200 μM DHEA for 24 h. Data represent mean ± standard deviation (SD) derived from three independent experiments. *p < 0.05; **p < 0.01; ***p < 0.001, compared to control (1% DMSO only) using *t-test*.

To examine the effect of DHEA on HNSCC cell viability, HNSCC cell lines including CAL 27, SAS, and HSC-3, were treated with different doses of DHEA for 24, 48, and 72 h, respectively. DHEA significantly inhibited cell viability in a time- and dose-dependent manner (**Figure 1A** and **Supplementary Figure 1A**). The half maximal inhibitory concentration (IC_50_) of DHEA was found to be 192.2 ± 28.4 μM for CAL 27 cells, 292.9 ± 43.9 μM for SAS cells, and 211.5 ± 13.5 μM for HSC-3 cells at 72 h. Also, we examined the effect of DHEA on the normal human oral fibroblast (HOF). As shown in **Figure 1B**, in contrast to HNSCC cell lines, CAL27 and SAS, DHEA 200 μM showed less toxicity and inhibitory effect in the viability of HOF after 72 h exposure. The cell viability decreased 20% in HOF, 32% in SAS and 50% in CAL 27. A previous study demonstrated that DHEA decreases the expression of stem cell genes in human sphere mesenchymal stem cells (38). Therefore, to examine the role of DHEA on HNSCC stemness potential, CAL 27 and SAS cells were incubated with 0, 100, and 200 μM DHEA for 20 days in a sphere formation assay. DHEA significantly suppressed the sphere size in both the HNSCC cell lines (**Figure 1C**). Furthermore, DHEA also decreased stemness-related mRNA levels, including *ALDH1A3*, *BMI-1*, *KLF4*, and *SOX2*, after 6 h of treatment in CAL 27 and SAS parental cells (**Figure 1D**) as well as in spheroid cells (**Figure 1E**). DHEA treatment resulted in a slight reduction in the protein expression of BMI-1 and Nestin but did not affect OCT4 and Nanog expression (**Figure 1F**). To further examine the transcriptional activity of stemness-related transcription factors, we used WNT (TCF/LEF), Nanog, OCT4, and Notch1 response element reporter assays. Although DHEA reduced the protein levels of OCT4 and Nanog only marginally, the transcriptional activities of OCT4 and Nanog, the stemness transcription factors, were markedly decreased in HNSCC cells (**Figure 1G**). Taken together, these results suggest that DHEA inhibits HNSCC cell viability and cancer stemness potential, including sphere size and expression of stemness markers.

### DHEA Inhibited Activity of the WNT Pathway by Decreasing Nuclear Translocation of Active β-Catenin

**Figure 2:**
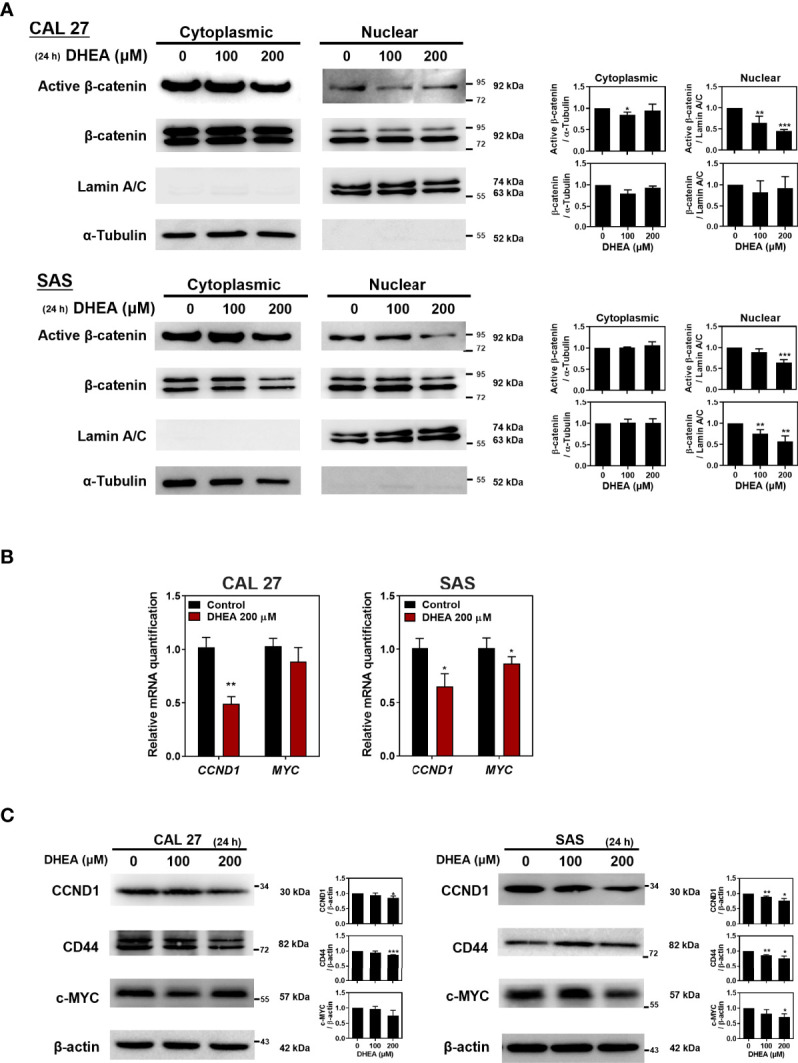
DHEA downregulated WNT pathway in HNSCC cells. **(A)** Western blotting with nuclear extracts showing the effect of treatment with 200 μM DHEA for 24 h on active β-catenin in CAL 27 and SAS cells. α-tubulin was used as a cytoplasmic control; lamin A/C was used as a nuclear fraction control. Active β-catenin was normalized to α-tubulin or lamin A/C. **(B, C)** RT-qPCR **(B)**, and western blotting **(C)** showing effect of DHEA on mRNA and protein expression of downstream genes of the WNT pathway in CAL 27 and SAS cells. *Left*, representative western blots of three independent experiments are shown. *Right*, bar charts represent the quantitation of three independent experiments. Data represent mean ± SD derived from three independent experiments. *p < 0.05; **p < 0.01; ***p < 0.001, compared to control (1% DMSO only) using *t-test*.

In human epithelial carcinomas, such as HNSCC or colorectal cancer, WNT signaling is crucial for the tumorigenesis and progression (44, 45). To further investigate the effect of DHEA on β-catenin, a crucial signal transducer of the WNT pathway, HNSCC cell lines were treated with DHEA followed by nuclear extraction assay. As shown in **Figure 2A**, DHEA treatment suppressed the nuclear translocation of active (non-phosphorylated) β-catenin, which prevented downstream effectors such as CCND1, CD44, and c-MYC (**Figures 2B, C**). Taken together, these results demonstrated that DHEA downregulates WNT transcriptional activity to inhibit the potential of HNSCC stemness.

### HNSCC Stem-Like Cells Elevated the Expression of the IRN Activity-Converting Enzyme CES1/2

**Figure 3:**
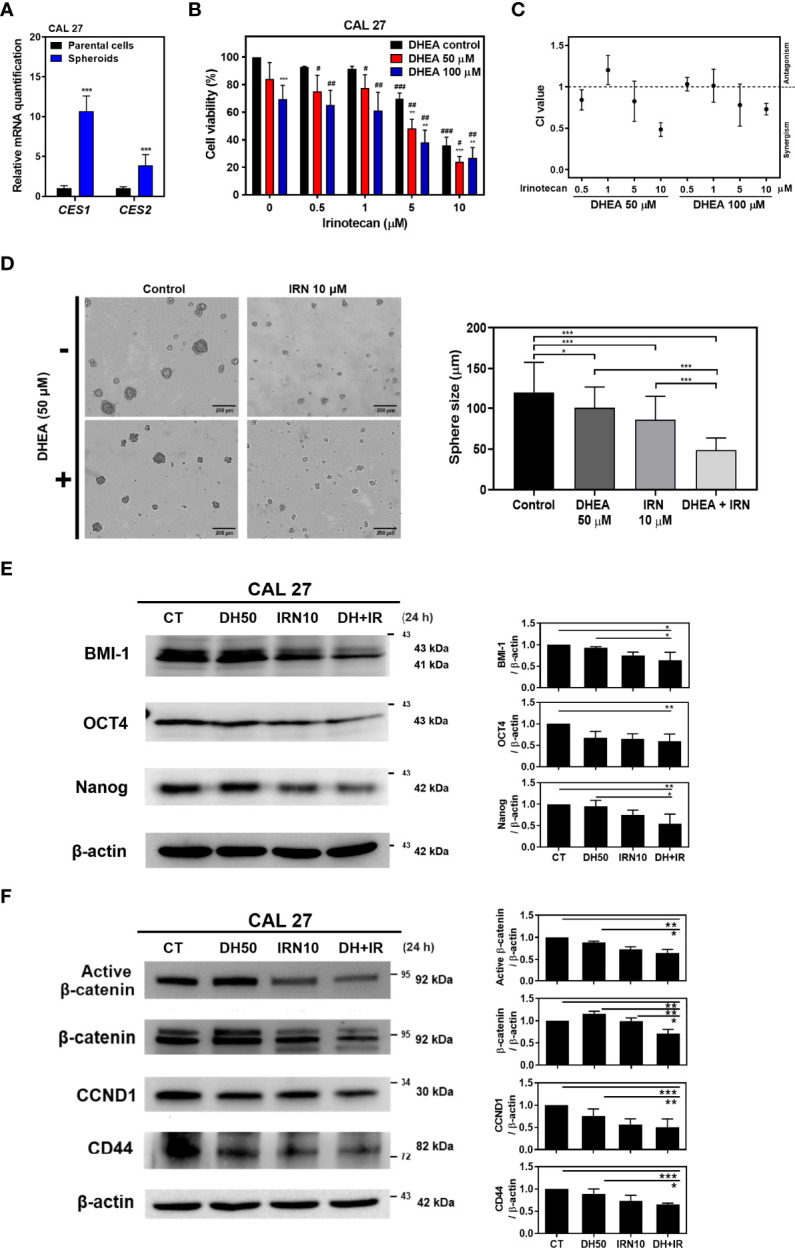
DHEA combinated with IRN synergistically decreased cell viability and stemness, and further downregulated WNT pathway in CAL 27. **(A)** RT-qPCR results showing mRNA level of IRN-metabolism enzymes in CAL 27 spheroids and parental cells. *CES1/2*: carboxylesterase 1/2. **(B)** CAL 27 cells were treated with DHEA (0, 50, and 100 μM), IRN (0, 0.5, 1, 5, and 10 μM) alone or in combination for 72 h and cell viability was determined by SRB assay. Data represent mean ± SD derived from three independent experiments. * *p* < 0.05; ** *p* < 0.01; *** *p* < 0.001, compared to the same concentration of DHEA using *t-test*. # *p* < 0.05; ## *p* < 0.01; ### *p* < 0.001, compared to the same concentration of IRN using *t-test.* **(C)** CI following various treatments in **(B)**. CI > 1, antagonism; CI = 1, additivity; CI < 1, synergism. **(D)** Sphere formation assay showing CAL 27 cells co-incubated with DHEA (50 μM) and/or IRN (10 μM). Scale bar: 200 µm **(E, F)** Western blotting showing protein expression of stemness markers **(E)**, and WNT pathway-related factors **(F)** in the whole cell lysate extracted from CAL 27 after DHEA and/or IRN treatment for 24 h *Left*, representative western blots of three independent experiments are shown. *Right*, bar charts represent the quantitation of three independent experiments. Data represent mean ± SD derived from three independent experiments. *p < 0.05; **p < 0.01; ***p < 0.001 using *t-test*. All treatments were maintained in the same percentage of DMSO. CT, control; DH, DHEA; IRN, irinotecan.

IRN, a topoisomerase I inhibitor, is a chemotherapeutic drug currently used for the treatment of colorectal cancer (46). In addition, IRN has been used in mono- and combination therapy along with other chemotherapeutic agents in patients with HNSCC and has shown improvement in patient response (18, 20). Following administration, IRN is converted to its active form, SN-38, by CES1/2 enzymes in patients (47). Recent studies have shown that the activity and expression of CES are related to IRN efficacy in lung cancer cell lines (17, 48) and solid tumors (49–51). In addition, Shaojun et al. demonstrated that in patients with metastatic colorectal cancer, high CES2 expression was correlated with better IRN therapeutic effect, which implies that CES2 may play an important role in IRN sensitivity. Therefore, evaluation of CES1/2 expression may provide preliminary clinical evidence for response to IRN-based therapies (16). Interestingly, CAL 27 spheroids showed higher *CES1/2* mRNA levels compared to their parental cells (**Figure 3A**). In addition, inhibition of WNT signaling decreased cancer stem cell-like features and increased the sensitivity of the cancer cells to chemotherapies, including IRN (52, 53). Our findings revealed that DHEA has an inhibitory effect on the WNT signaling pathway (**Figures 1F** **and** **2**). Hence, we sought to determine whether DHEA sensitizes HNSCC CSCs to IRN.

### DHEA Combined With IRN Showed Improved Anti-Cancer as Well as Anti-Stemness Potential Effect, and Further Downregulated WNT Pathway in HNSCC Cells

To evaluate the effect of DHEA combined with IRN on HNSCC, cell viability was examined using SRB assay and the synergistic effect was assessed *via* combination index (CI) calculation by using CompuSyn software (39). In CAL 27 and HSC-3 cells, DHEA combined with IRN further inhibited cell viability compared to DHEA or IRN alone at 72 h treatment, and the CI index showed a synergistic effect (CI value < 1) (**Figures 3B, C** and **Supplemental Figures 1B, C**). The viability of CAL 27 cells following DHEA (50 μM), IRN (10 μM), and combination treatment was 84.1%, 35.9%, and 24.0%, respectively. This dose combination showed the best synergism (CI value = 0.48) and was used to perform subsequent experiments in CAL 27 cells. In addition, other chemotherapeutic agents were tested in combination with DHEA in CAL 27 and SAS cells (**Supplemental Figure 2**). Some of the combination treatments of DHEA plus gemcitabine, docetaxel or methotrexate showed synergistic effect but less than that of DHEA plus IRN. Among these chemotherapeutic drugs, docetaxel or gemcitabine obtained quite effective single agent chemotherapy in HNSCC treatment cells. Therefore, we did not further examine the combination uses to enhance cytotoxicity. Therefore, IRN was selected as the combination chemotherapeutic drug with DHEA for further studies. In the sphere formation assay, the combination significantly decreased CAL 27 sphere size compared to DHEA or IRN alone (**Figure 3D**). In addition, the combination treatment showed a greater inhibitory effect on the expression of stemness markers, including BMI-1, OCT4, and Nanog (**Figure 3E**). Notably, in the WNT pathway, the combination treatment further decreased the expression of active non-phosphorylated β-catenin and downstream targets, such as CCND1 and CD44 from the whole cell lysate (**Figure 3F**). These data confirmed that combination treatment of DHEA with IRN exerted better anticancer and stem cell like traits inhibitory effects compared to DHEA or IRN alone in HNSCC cells.

### DHEA Combined With IRN Showed Better Anti-Tumor Effect Than IRN Monotherapy in Subcutaneous HNSCC Mouse Models

**Figure 4:**
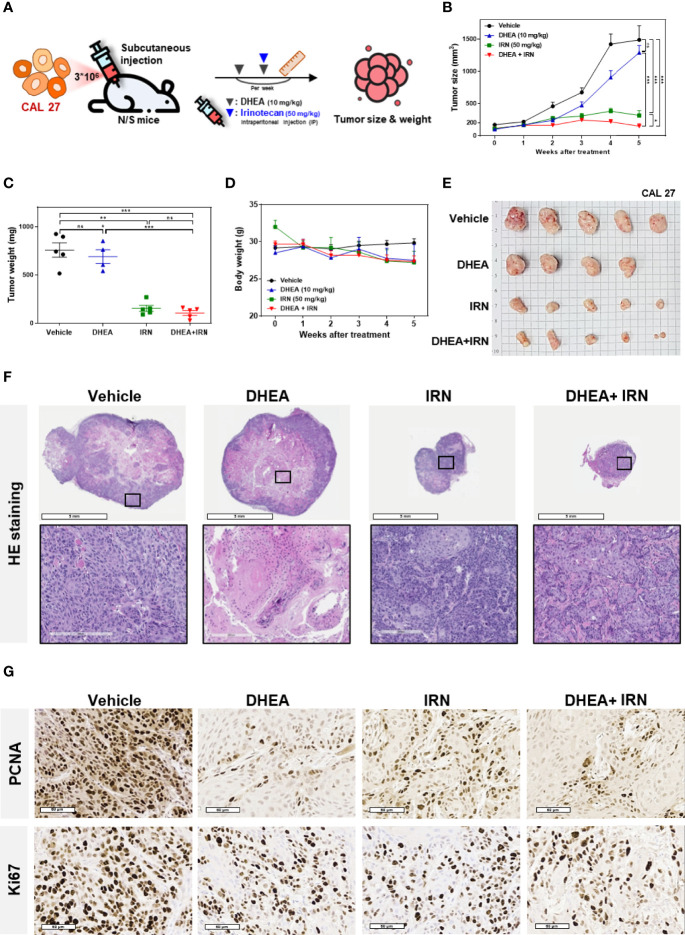
DHEA combined with IRN showed increased anti-tumor effect in HNSCC subcutaneous mouse models. **(A)** Flow chart showing the experimental schedule and drug administration. CAL 27 cells were subcutaneously injected into mice and then treated with DHEA (10 mg/kg/twice a week) and IRN (50 mg/kg/once a week) *via* intraperitoneal injections. Tumor size and body weight were measured once a week. **(B–E)** Tumor size **(B)**, tumor weight **(C)**, body weight **(D)**, and tumor appearance **(E)** of CAL 27 tumor-bearing mice in vehicle- and drug-treated groups. **(F)** Hematoxylin and eosin (H&E) staining results of tumors from mice following vehicle and drug treatment. **(G)** IHC staining for PCNA (*upper*) and Ki67 (*lower*) in tumors from mice following various treatments. Data represent mean ± SEM (*n* = 5/per group). *p < 0.05; **p < 0.01; ***p < 0.001, *n.s.* not significant, compared to each other using *t-test*. IRN, irinotecan.

To further investigate the effect of DHEA combined with IRN against HNSCC *in vivo*, CAL 27 cells were subcutaneously injected into the flank of immunodeficient mice to establish xenograft models. DHEA (10 mg/kg/twice a week) and/or IRN (50 mg/kg/once a week) were administered *via* intraperitoneal injection (**Figure 4A**). Compared to DHEA or IRN alone, the combination treatment showed greater inhibitory effect on tumor size and weight compared to the controls (**Figures 4B, C, E**). Interestingly, the combination treatment alleviated irinotecan-induced loss of body weight, suggesting that DHEA may reduce the side effects of IRN (**Figure 4D**). In H&E staining of HNSCC xenografts (**Figure 4F**), the tumor size of the IRN alone and the combination treatment groups was smaller than that of vehicle or DHEA groups. As shown in **Figure 4G**, mice with combination treatment showed lower expression of the proliferation markers PCNA and percentage of Ki67, compared to those treated with drug alone. Taken together, DHEA enhanced irinotecan-mediated anticancer effects and further reduced tumorigenicity *in vivo*.

### DHEA Combined With IRN Exerted Better Anti-Tumor Effect Than IRN Monotherapy in Orthotopic Mice Models

**Figure 5:**
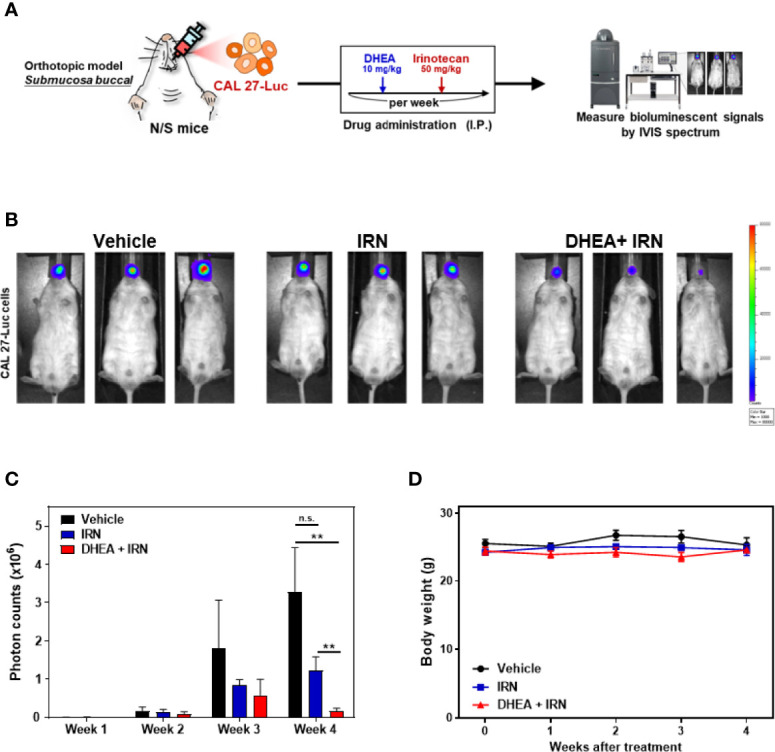
DHEA combined with IRN further reduced tumor growth in HNSCC orthotopic mouse models. **(A)** Flow chart of the experimental schedule and drug administration. CAL 27 cells with luciferase expression (CAL 27-Luc) were injected into the buccal submucosa of mice. DHEA (10 mg/kg/once a week) and IRN (50 mg/kg/once a week) were injected intraperitoneally (IP). Tumor growth (measured by bioluminescent signals using the IVIS Spectrum Imaging System) and body weights were measured once a week. **(B-D)** Bioluminescence images **(B)**, quantitation of photon counts **(C)**, and body weights **(D)** of orthotopic mouse models following various treatments. Data represent mean ± SEM (*n* = 5/per group). *n.s.* not significant; **p < 0.01, compared to each other using t-test .

To further evaluate the efficacy of DHEA in an orthotopic oral cancer model, CAL 27 cells with luciferase (Luc)-expression were inoculated into the buccal submucosa of immunodeficient mice. The drugs were injected intraperitoneally, and the tumor growth rate was assessed by measuring the bioluminescence signals using the IVIS Spectrum System once a week. Treatment with DHEA alone did not have a significant effect on inhibition of tumor growth in the orthotopic oral cancer model (**Supplemental Figure 3**). The CD44^+^/CD133^+^ CAL 27 stem-like cells *in vivo* models revealed that DHEA treatment reduced the HNC stem-like cells’ tumor formation frequency more than control (**Supplemental Table 4**). To further investigate the effect of DHEA combined with IRN in the orthotopic oral cancer model, mice were separated into three groups for different treatments: vehicle, IRN (50 mg/kg once a week), and DHEA (10 mg/kg/once a week) combined with irinotecan (**Figure 5A**). As shown in **Figures 5B, C**, DHEA combined with IRN caused a significant reduction in the orthotopic HNSCC xenograft bioluminescent signals in the buccal sites of the mice compared to vehicle or IRN alone. Taken together, these results revealed that compared to monotherapy, DHEA combined with IRN demonstrated increased anti-tumor effect in the orthotopic mouse model of oral cancer.

## Discussion

In the present study, we found that DHEA showed anticancer and cancer stem cell-like traits of HNSCC cells *via* downregulation of the WNT signaling pathway (**Figures 1**, **2**). In the drug combination treatment strategy, DHEA plus IRN exerted a synergistic effect by further reducing cell viability, inhibiting cancer stem cell-like features, and suppressing WNT signaling *in vitro* (**Figure 3**). Furthermore, the combination treatment showed better anti-tumor growth effect in subcutaneous and orthotopic mouse models (**Figures 4**, **5**).

DHEA is the most abundant steroid in human serum at young age and is a precursor for the sex hormones. DHEA has been used as a dietary supplement and reported to show anti-aging and anti-inflammatory effects. Recently, using the drug repurposing approach, DHEA has been studied in various diseases, including cancer. Several studies have demonstrated that DHEA affects cancer *via* various signaling pathways. In breast cancer, several reports have demonstrated that DHEA inhibits the metastatic processes, including cell migration, invasion, and anchorage-independent growth, and partially reverses the EMT process, and suppresses tumor growth in MDA-MB-231-mouse xenografts (30–32). In hepatoma, DHEA inhibits PI3K/AKT signaling to induce apoptosis and thereby decreases cell proliferation in HepG2 cells (35). In colon cancer, DHEA shows anticancer proliferation *via* induction of cell cycle arrest in the G0/G1 phase in HT-29 cells (35). In myeloma, DHEA decreases cell number and induces expression of *PPARβ* and *IκBα* genes *via* downregulation of interleukin-6 (33). In pancreatic cancer, DHEA administration significantly suppressed tumor growth *in vivo* by altering plasma sex hormone concentrations (36). These findings suggest that DHEA affects many aspects of cancer cells, but some potential functions and important roles of DHEA have never been explored. In this study, we investigated the effect of DHEA on HNSCC and cancer stemness. Our findings demonstrated that DHEA had an inhibitory effect on HNSCC viability and was less toxic to normal cells, HOF (**Figures 1A, B**).

CSCs are a major obstacle in effective cancer treatment due to their self-renewal capability. Previously, DHEA has been reported to suppress the expression of stem cell genes, including *SOX2, Nanog*, and *OCT4* (38) in human sphere mesenchymal stem cells. Furthermore, DHEA has been found to decrease the spheroid size of breast cancer, which may have the potential to suppress cancer stemness (30). However, the effect of DHEA on cancer stemness-related events and the underlying mechanisms have never been studied. Our results showed that DHEA suppressed cancer stemness properties of HNSCC, including decreased sphere size and transcriptional activities of stemness-related transcription factors, such as WNT (TCF/LEF), Nanog, and OCT4. OCT4 and Nanog are pluripotent transcriptional factors that contribute to maintenance of stemness and cancer progression (54, 55). Although the expression of OCT4 and Nanog was slightly decreased following DHEA treatment, their transcriptional activities were significantly decreased by DHEA, indicating that DHEA has the ability to inhibit CSC potential (**Figures 1C-G**).

One possible strategy to overcome with the ineffectiveness of cancer chemotherapies is to target the key signaling pathways that promote cancer stemness. The WNT/β-catenin signaling pathway regulates the maintenance and self-renewal of CSCs in colon cancer (56) and breast cancer (57), and shows significantly higher activation in breast CSCs compared to that in normal stem-like cells (58). Aberrant activation of the WNT signaling pathway has already been demonstrated in HNSCCs and CSCs (59). Binding of the WNT ligands to the WNT receptors activates the WNT pathway, accumulation and translocation of stable, non-phosphorylated β-catenin into the nucleus and binding to the TCF/LEF transcription factors. Consequently, the activation of transcription factors initiates the expression of downstream target genes. Thus, blocking β-catenin, a key factor in the WNT pathway, may be an effective strategy for inhibiting the WNT pathway (60). Several studies have developed WNT pathway inhibitors, including targeting β-catenin transcriptional activity and β-catenin target genes (14). However, in clinical trials among patients with HNSCC, WNT974, a Porcupine (PORCN) inhibitor that blocks the secretion of WNT ligands, is the only drug being administered (14, 61). These findings suggest that targeting WNT/β-catenin signaling represents a promising therapeutic strategy for HNSCC. DHEA inhibited WNT signaling *via* downregulation of active β-catenin in the nucleus, thereby decreasing the transcriptional activity of downstream target genes, such as *CD44* and *CCND1* in HNSCC (**Figure 2**). Previously, Li et al. found that DHEA prevents osteoarthritis by regulating the WNT/β-catenin pathway and decreasing the expression of β-catenin (62). Our observations are the first to reveal the mechanism of DHEA on β-catenin within the WNT pathway in cancers, especially in HNSCC.

Chemoresistance of CSCs causes failure of cancer therapy and tumor recurrence (4). Accumulating evidence suggests that agents that block the WNT pathway may sensitize cancer cells and CSCs to chemotherapies and may serve as novel synergistic therapeutic regimens in combination treatment strategies (63). As our data showed that DHEA inhibited cancer stem cell-like traits *via* downregulation of the WNT pathway in HNSCC, we further applied DHEA to combination therapy. In addition, mRNA levels of *CES1/2*, which encode the enzymes involved in generation of the active form of IRN, were higher in CAL 27 spheres than in their parental cells (**Figure 3A**). This hint that the spheres may be more sensitive to IRN than parental cells due to their higher CESs. DHEA combined with IRN exerted a synergistic effect on cell viability, and the most optimal dose was found to be 50 μM DHEA plus 10 μM IRN as revealed *via* CI index calculation (**Figures 3B, C**). We also examined other chemotherapeutic drugs in combination with DHEA, but their anticancer effects were lesser than that of IRN (**Supplemental Figure 2**). Furthermore, compared to DHEA or IRN alone, the combination treatment further downregulated the sphere size, expression of proteins associated with stemness, as well as the WNT pathway in HNSCC cells (**Figure 3**). Moreover, DHEA plus IRN demonstrated inhibitory effect on tumor growth in a subcutaneous and an orthotopic oral cancer model (**Figures 4**, **5**). Although we used a general subcutaneous HNSCC mice model rather than the classical cancer stem cells-based animal models by serially diluted inoculation to investigate the effect of DHEA and/or IRN on tumor inhibition. However, Shrivastava et al. have observed CAL 27 cells possessed about 1.6% of CSC population in total number of parental cells (64). In our subcutaneous *in vivo* models, there were about 48,000 CSCs among the inoculation. This subset of cells might mimic the ability of CSCs to tumor initiation and progression and provide the preliminary result about the inhibitory effect of DHEA on CSC potential. In additional, in our study, the 10 mg/kg DHEA used in the mice administration was converted from a human equivalent dose (50 mg) based on body surface area by the formula from the US Food and Drug Administration and from Chen et al. study (65). Also, as shown in our results, there were no abnormal change of body weight of mice or other side effects observed, suggesting that this dosage was tolerable. In addition, the acute oral toxicity (lethal dose, LD50) of DHEA is >10,000 mg/kg in mouse, further supporting that there is no acute toxicity of DHEA. Although the underlying mechanism of the DHEA-mediated anticancer effect of IRN needs to be further elucidated, the *in vitro* and *in vivo* data presented in this study provide evidence supporting the synergistic effect of DHEA and IRN against HNSCC.

## Conclusions

Taken together, our findings indicate that DHEA exerts anticancer effects, especially with regard to the inhibitory effect of cancer stem-like cells, *via* downregulation of the WNT pathway *in vitro* and reduces tumorigenicity *in vivo.* Furthermore, DHEA enhances the therapeutic efficacy of IRN against HNSCC cells. The combination treatment showed increased tumor growth inhibition in both subcutaneous and orthotopic mouse models. These results highlight the need for more in-depth investigations to understand the underlying mechanism associated with the synergistic effects of DHEA and IRN. Our results provide a novel and promising therapeutic strategy for patients with HNSCC.

## Data Availability Statement

The raw data supporting the conclusions of this article will be made available by the authors, without undue reservation.

## Ethics Statement

The animal study was reviewed and approved by Institutional Animal Care and Use Committee of Academia Sinica (Taipei, Taiwan, protocol no.: ASIACUC-R19-07-1329.

## Author Contributions

Conception and design: L-JL, PC, W-MC, and C-YH; Development of methodology: L-JL and C-YY; Acquisition of data (provided animals, provided facilities, etc.): PC, MH, and C-YH; Analysis and interpretation of data (e.g., statistical analysis, biostatistics, computational analysis): L-JL, C-YY, and S-WF; Writing, review, and/or revision of the manuscript: L-JL, C-HL, T-CL, and PC; Funding: MH, C-YH, and PC; Study supervision: W-MC and C-YH; All authors contributed to the article and approved the submitted version.

## Funding

This study was financially supported from Genomics Research Center (MH), from the Ministry of Science and Technology (MOST107-3011-B-010-001-, MOST107-2320-B-010-040-MY3, MOST109-2320-B-010-026-, and MOST110-2320-B-A49A-541- to CYFH, and MOST110-2320-B-075-009, 109-2320B-075-003, 109-2314B-075-080 to PMHC) and from Taipei Medical University (111TMU-TMUH-03-2 to WMC).

## Conflict of Interest

The authors declare that the research was conducted in the absence of any commercial or financial relationships that could be construed as a potential conflict of interest.

## Publisher’s Note

All claims expressed in this article are solely those of the authors and do not necessarily represent those of their affiliated organizations, or those of the publisher, the editors and the reviewers. Any product that may be evaluated in this article, or claim that may be made by its manufacturer, is not guaranteed or endorsed by the publisher.

## Abbreviations

CES1/2, carboxylesterases1/2; CSCs, cancer stem cells, HNSCC, head and neck squamous cell carcinoma; IRN, irinotecan; DHEA, dehydroepiandrosterone; SRB, sulforhodamine B; TCF/LEF, T cell factor/lymphoid enhancer factor.
