# Supplementary figures and images for: Dehydroepiandrosterone (DHEA) Sensitizes Irinotecan to Suppress Head and Neck Cancer Stem-Like Cells by Downregulation of WNT Signaling
Source: Front Oncol. 2022 Jul 13;12:775541. doi: 10.3389/fonc.2022.775541 (PMC9328800; doi:10.3389/fonc.2022.775541)

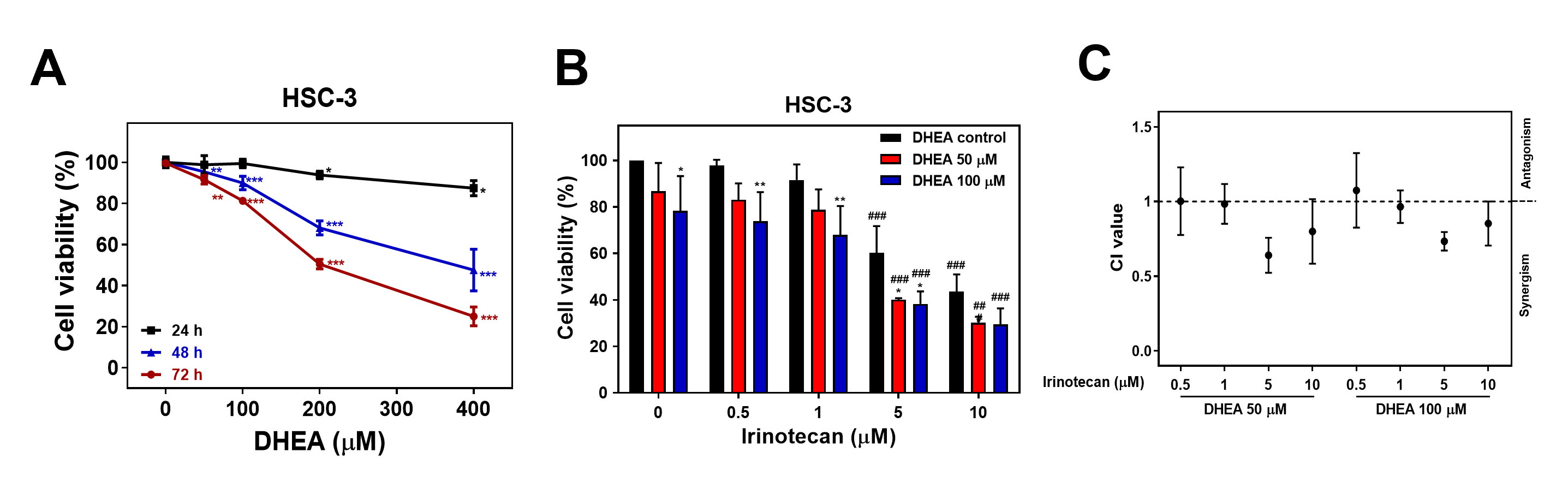

Supplement: Supplementary file 1 [file Image_1.tif]

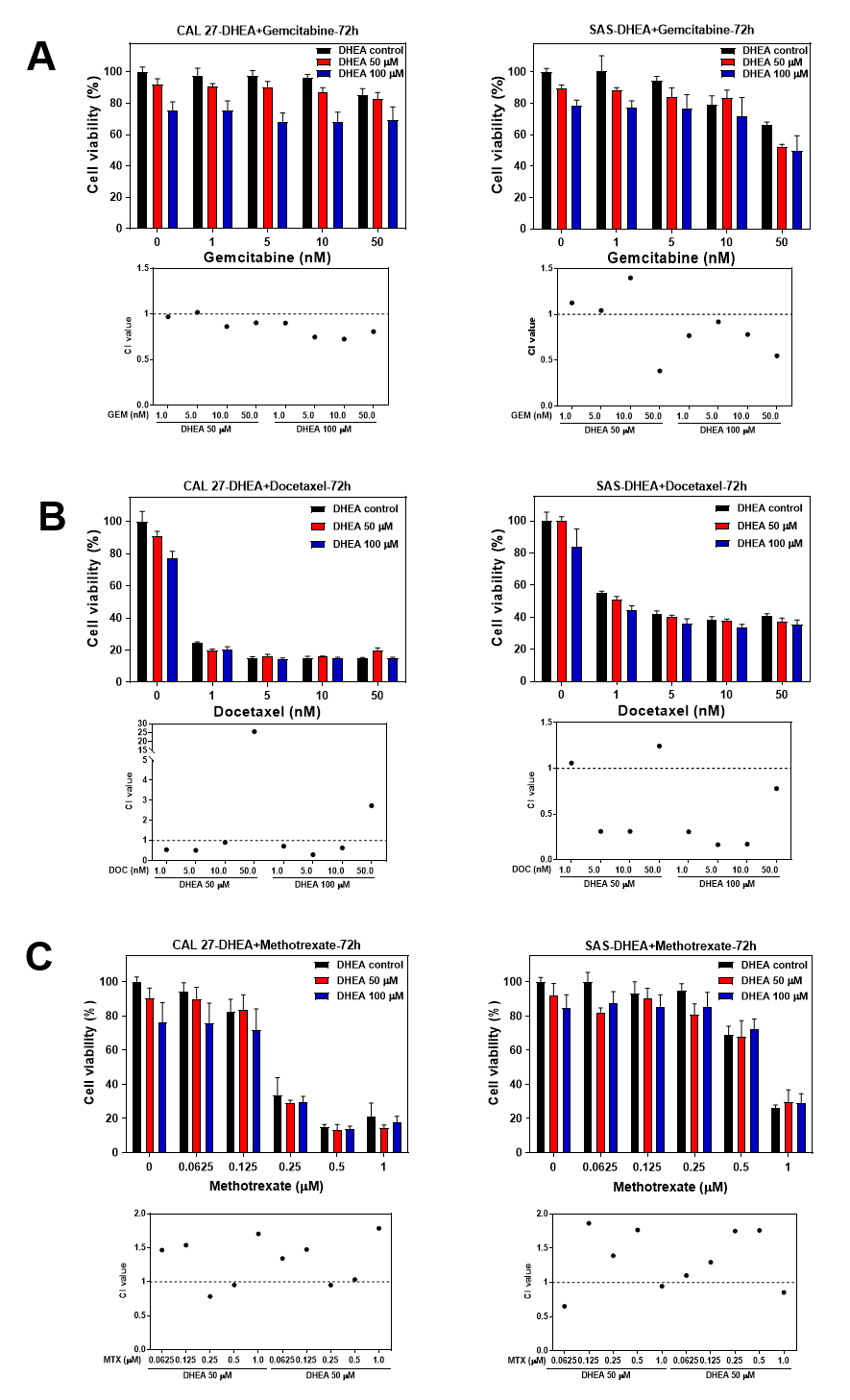

Supplement: Supplementary file 2 [file Image_2.tif]

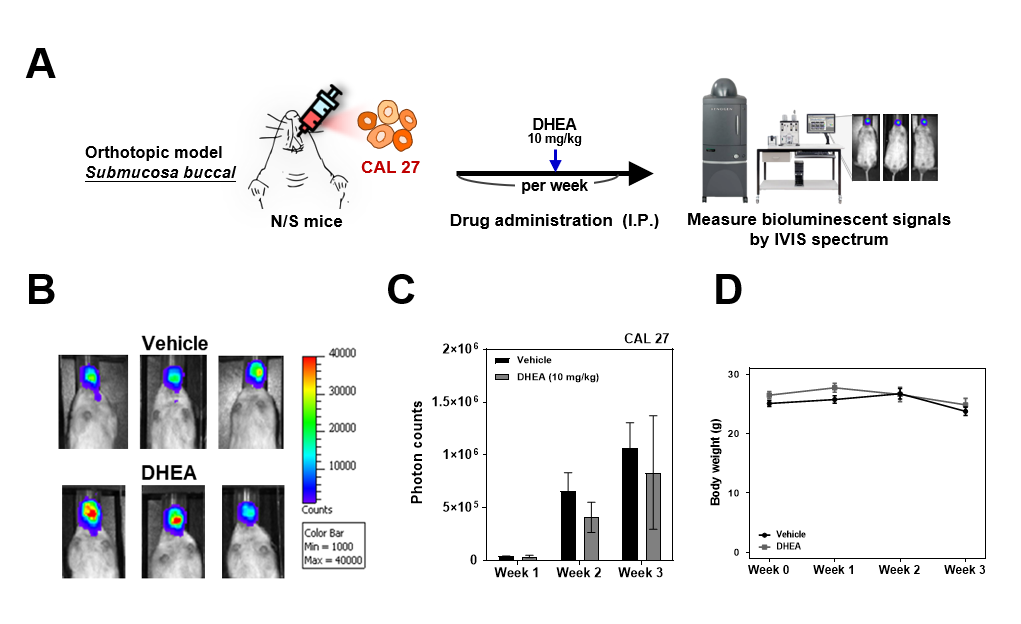

Supplement: Supplementary file 3 [file Image_3.tif]
