# Supplementary material for: Dehydroepiandrosterone (DHEA) Sensitizes Irinotecan to Suppress Head and Neck Cancer Stem-Like Cells by Downregulation of WNT Signaling
Source: Front Oncol. 2022 Jul 13;12:775541. doi: 10.3389/fonc.2022.775541 (PMC9328800; doi:10.3389/fonc.2022.775541)
Supplement: Supplementary file 4 [file Table_1.docx]

**Supplementary Table 1. Chemicals, reagents, kits, and constructs.**

| **Chemicals** | **Company** | **Catalog number** |
| --- | --- | --- |
| Trans-dehydroepiandrosterone (DHEA) | Sigma | D4000 |
| Irinotecan | Sigma | I1406 |
| Trichloroacetic acid | Sigma | T8657 |
| Sulforhodamine B (SRB) | Sigma | S1402 |
| Basic FGF | PeproTech | 100-18B |
| EGF | PeproTech | AF-100-15 |
| 50X B27 supplement | Gibco | 17504044 |
| Immobilon western chemiluminescent HRP substrate | Millipore | WBKLS0500 |
| Polybrene | Sigma | H9268 |
| Puromycin | Invitrogen | A1113803 |
| ONE-Glo Luciferase Assay System | Promega | E6120 |
| Bradford assay | Bio-Rad | 5000006 |
| SuperScript III kit | Invitrogen | 18080051 |
| TRIzol™ Reagent | Invitrogen | 15596026 |
| **Constructs** | **Company** | **Catalog number** |
| pGreenFire1-TCF/LEF (EF1α-puro) Lentivector | System Biosciences | TR013PA-P |
| pGreenFire1-Nanog (EF1α-puro) Lentivector | System Biosciences | TR019PA-P |
| pGreenFire1-Oct4 (EF1α-puro) Lentivector | System Biosciences | TR039PA-P |
| pGreenFire1-Notch (EF1α-puro) Lentivector | System Biosciences | TR020PA-P |
