# Supplementary material for: Dehydroepiandrosterone (DHEA) Sensitizes Irinotecan to Suppress Head and Neck Cancer Stem-Like Cells by Downregulation of WNT Signaling
Source: Front Oncol. 2022 Jul 13;12:775541. doi: 10.3389/fonc.2022.775541 (PMC9328800; doi:10.3389/fonc.2022.775541)
Supplement: Supplementary file 5 [file Table_2.docx]

**Supplementary Table 2. Antibodies list.**

| **Target** | **Company** | **Catalog number** | **MW (kDa)** | **Dilution /2Ab** |
| --- | --- | --- | --- | --- |
| BMI-1 | Novus Biologicals | NBP1-33748 | 43, 41 | 1:1000 R |
| Nanog | Cell Signaling Technology | CST#4903 | 42 | 1:1000 R |
| OCT4 | Abcam | Ab19857 | 43 | 1:1000 R |
| Nestin | Boster | M00806 | 177 | 1:1000 M |
| β-catenin | Cell Signaling Technology | CST#9562 | 92 | 1:1000 R |
| Non-p-β-catenin (Ser33/37/Thr41) | Cell Signaling Technology | CST#8814 | 92 | 1:1000 R |
| CCND1 | Boster | M00149-1 | 30 | 1:1000 R |
| CD44 | Boster | M00052-1 | 82 | 1:1000 R |
| c-MYC | Cell Signaling Technology | CST#5605 | 57 | 1:1000 R |
| β-actin | Sigma | A5316 | 42 | 1:5000 M |
| α-tubulin | Sigma | 035M4878V | 52 | 1:5000 M |
| Lamin A/C | Boster | M00438 | 74, 63 | 1:1000 R |
| PCNA | GeneTex | 100539 | *IHC | 1:500 |
| Ki67 | Dako | M7240 | *IHC | 1:150 |
| Abbreviations: 2Ab, secondary antibody; R, rabbit; M, mouse. *IHC: antibody was used in IHC staining. | | | | |
