# Supplementary material for: Dehydroepiandrosterone (DHEA) Sensitizes Irinotecan to Suppress Head and Neck Cancer Stem-Like Cells by Downregulation of WNT Signaling
Source: Front Oncol. 2022 Jul 13;12:775541. doi: 10.3389/fonc.2022.775541 (PMC9328800; doi:10.3389/fonc.2022.775541)
Supplement: Supplementary file 6 [file Table_3.docx]

**Supplementary Table 3. Primer sequences.**

| **Gene** | **Primer sequence (5’ – 3’)** | **Gene** | **Primer sequence (5’ – 3’)** |
| --- | --- | --- | --- |
| ALDH1A3-F627-47 | ACCTCTCACCGCCCTTTATCT | GAPDH-R | GAAGATGG GATGGGATTTC |
| ALDH1A3-R767-46 | GTGAAGGCGATCTTGTTGATCT | KLF4-F1549-68 | ACCCTGGGTCTTGAGGAAGT |
| BMI1-F | TGGAGAAGGAATGGTCCACTTC | KLF4-R1706-85 | GGCATGAGCTCTTGGTAATGGA |
| BMI1-R | GTGAGGAAACTGTGGATGAGGA | MYC-F1057-76 | TCTCCGTCCTCGGATTCTCT |
| CCND1-F | GACCTTCGTTGCCCTCTGT | MYC-R1179-58 | TTCTTGTTCCTCCTCAGAGTCG |
| CCND1-R | TGAGGCGGTAGTAGGACAGG | NANOG-F611-31 | ACCTCAGCTACAAACAGGTGA |
| CES1-F | ACCCCTGAGGTTTACTCCACC | NANOG-R717-98 | CTTCTGCGTCACACCATTGC |
| CES1-R | TGCACATAGGAGGGTACGAGG | OCT4-F1232-53 | GGGGTTCTATTTGGGAAGGTAT |
| CES2-F | CATGGCTTCCTTGTATGATGGT | OCT4-R1358-39 | TGTTGTCAGCTTCCTCCACC |
| CES2-R | CTCCAAAGTGGGCGATATTCTG | SOX2-F1103-23 | TACAGCATGTCCTACTCGCAG |
| GAPDH-F | GAAGGTGAAGGTCGGAGT | SOX2-R1212-92 | GAGGAAGAGGTAACCACAGGG |
