# Supplementary material for: Dehydroepiandrosterone (DHEA) Sensitizes Irinotecan to Suppress Head and Neck Cancer Stem-Like Cells by Downregulation of WNT Signaling
Source: Front Oncol. 2022 Jul 13;12:775541. doi: 10.3389/fonc.2022.775541 (PMC9328800; doi:10.3389/fonc.2022.775541)
Supplement: Supplementary file 7 [file Table_4.docx]

**Supplementary Table 4. The *in vivo* tumor formation frequency of CAL 27 stem-like cells with DHEA treatment.**

| **Treatment** | **Tumor formation frequency** |
| --- | --- |
| **Control** | **8/10** |
| **DHEA 10 mg/kg** | **4/9** |
